# Cultural Humility: The Bias Checker

**DOI:** 10.1093/geroni/igab046.472

**Published:** 2021-12-17

**Authors:** Natalie Moore-Bembry

**Affiliations:** Rutgers, The State University of New Jersey, Camden, New Jersey, United States

## Abstract

Historically we have been taught to understand and embrace cultural competency, however, this focus has often led to a superficial understanding of others and seldom required one to better understand themselves. Cultural humility is based on one’s ability to engage in individual accountability and institutional accountability. Individual accountability is based on critical self-reflection and critique, lifelong learning, and the challenging of power imbalances. Institutional accountability requires one to challenge structural power. This session will: (1) explore ways to engage in critical self-reflection and critique; (2) describe how values and beliefs impact the interactions of our personal and professional lives; and (3) strategize ways to collectively model and practice in cultural humility in one’s personal and professional life.

